# Decoupling Distribution of *n*-Alkanes in Aeolian Sand and Vegetation of the Northern Ulan Buh Desert, China: Insight into Organic Matter Preservation in Arid Regions

**DOI:** 10.3390/plants13202898

**Published:** 2024-10-17

**Authors:** Shangzhe Zhou, Lei Xi, Mengchun Cui, Guipeng Cui, Pan Gao, Jinlei Zhu, Weiyuan Kong, Yufu Jia, Qi Lu

**Affiliations:** 1 Institute of Ecological Conservation and Restoration, Chinese Academy of Forestry, Beijing 100091, China; shangzhe@caf.ac.cn (S.Z.); xilei@caf.ac.cn (L.X.); cuimc@caf.ac.cn (M.C.); cuigp@caf.ac.cn (G.C.); pangao@caf.ac.cn (P.G.); jinleizhu@caf.ac.cn (J.Z.); luqi@caf.ac.cn (Q.L.); 2 Institute of Desertification Studies, Chinese Academy of Forestry, Beijing 100091, China; 3 Institute of Great Green Wall, Beijing 100091, China

**Keywords:** *n*-alkanes, desert plants, aeolian sands, Ulan Buh Desert, climate reconstruction

## Abstract

Fallen leaves and their decomposition directly deposit leaf wax *n*-alkanes into sediments, which can be used to identify local flora. These *n*-alkanes are important for studying past vegetation and climate, but their distribution in sediments must be known. Aeolian sand *n*-alkanes are particularly important for understanding paleoclimates in arid regions, despite the challenges of extraction due to their extremely low abundance. To investigate the preservation of plant leaf wax *n*-alkanes in deserts, we analyzed *n*-alkanes in aeolian sands from the Northern Ulan Buh Desert (UBD), China, and compared them to the surrounding vegetation. We calculated the total *n*-alkane concentration (ΣALK), average chain length (ACL_21–35_), and carbon preference index (CPI_21–35_). In the Northern UBD, aeolian sand *n*-alkanes have lower ΣALK, indicating microbial degradation. The eastern aeolian sand has lower CPI_21–35_ and ACL_21–35_ than the adjacent vegetation, whereas the western sand values are consistent with the plants, likely due to the transport of plant-derived materials by wind and water from the nearby mountains. Our study shows that sedimentary *n*-alkane signatures are not only determined by local vegetation but also influenced by environmental factors like temperature and precipitation. Additionally, local deposition processes play a significant role in determining the properties of these *n*-alkanes.

## 1. Introduction

Leaf waxes are important plant-derived compounds that coat the leaf surface and play a vital role in reducing water evaporation, protecting against ultraviolet radiation, and preventing microbial infections [1,2,3,4,5]. Long-chain *n*-alkanes (*n*-C_27_–*n*-C_35_) are the main components of plant leaf waxes, with odd-numbered carbon chain length *n*-alkanes generally being more abundant than even-numbered ones [1,6,7]. In addition to leaves, many other plant organs can synthesize long-chain *n*-alkanes with an odd-over-even carbon number preference, but their concentrations are much lower than those found in leaf waxes [8,9,10].

Once plants die or their leaves decay and are incorporated into sedimentary systems, leaf wax *n*-alkanes are transformed into components of sedimentary *n*-alkanes [11,12]. These compounds preserve the structural legacy of their botanical origins, rendering them indispensable biomarkers for deciphering ancient vegetation and climatic conditions [13,14,15,16,17]. However, soil formation, diagenesis, and sedimentation processes can modify and complicate the information recorded by the *n*-alkanes in sedimentary systems over time and across space [18,19]. Therefore, comprehending the temporal and spatial dynamics of sedimentary *n*-alkanes is essential.

Due to their low concentrations, leaf wax *n*-alkanes in the litter are readily dispersed by wind or transported by water. Therefore, sedimentary leaf wax *n*-alkanes typically reflect the collective input from the surrounding plant community, rather than being exclusive to specific local plant species [20,21]. Wind-blown *n*-alkane molecules are capable of traveling long distances and settling with atmospheric particulate matter, along with precipitation [18,22,23,24,25]. On the other hand, water transports *n*-alkane molecules over shorter distances than wind, via rivers or runoff, by carrying fallen leaves or sediments [18,26]. The variations in transportation and deposition processes can result in the accumulation of sedimentary leaf wax *n*-alkanes, potentially originating from either regional plant communities or specific local plant populations [10]. This diversity in sources can influence the composition and interpretation of *n*-alkane signatures in sedimentary records.

Organic biomarkers, including *n*-alkanes, are generally believed to be well preserved in desert environments due to the minimal microbial activity [27]. Thus, the arid regions seem like ideal areas for the study of paleovegetation and paleoclimate based on organic biomarkers. In reality, the scarce vegetation in desert regions caused by arid conditions, combined with environmental factors such as strong winds, leads to difficulties in preserving organic matter and results in low organic content in the aeolian sands. This situation undoubtedly escalates the challenges of using organic biomarkers to reconstruct paleoclimates in desert environments. Previous studies have documented differences in *n*-alkane compositions between desert plants and soils in extremely arid environments such as the hyperarid Atacama Desert and the cold and arid Gurbantunggut Desert, which might be constrained by abiotic factors like precipitation [28,29]. However, a definitive explanation for these discrepancies has yet to be agreed upon.

Given this, we examined the *n*-alkane signatures in aeolian sands from the Eastern Yamalik Desert (YD) and the Northern Ulan Buh Desert (UBD) and plants from the Northern UBD, which is located on the edge of the East Asian Monsoon Region. This makes it an ideal location for studying the evolution of paleoclimate in deserts across China [30,31,32]. Our research aims to (1) ascertain whether the aeolian sands of the desert accurately mirror the *n*-alkanes signatures of the surrounding desert vegetation and (2) clarify the sources and preservation mechanisms of the sedimentary *n*-alkanes in the Northern UBD. This research will provide a benchmark for future endeavors in paleoclimate reconstruction in desert environments, offering valuable insights into the dynamics of organic matter—how it moves, is transported, and ultimately settles through the complex processes of aeolian sedimentation.

## 2. Results

### 2.1. Aeolian Sand n-Alkane Abundances and Distribution Patterns

To characterize the abundances and distribution patterns of aeolian sand *n*-alkanes, the total concentration of *n*-alkanes (ΣALK), the proportion of short-chain (*n*-C_16_–*n*-C_20_) *n*-alkanes (Σ_16–20_/ΣALK), the proportion of long-chain (*n*-C_27_–*n*-C_35_) *n*-alkanes (Σ_27–35_/ΣALK), the carbon number of long-chain *n*-alkanes with the highest abundance (C_MAX_), the average chain length (ACL_21–35_), and the carbon preference index (CPI_21–35_) are calculated and shown in Table 1, and the detailed calculation method is described in Section 4.3. The UBDNE, UBDNW, UBDSW, UBDSE, and UBDC represent the aeolian sand samples from the northeastern, northwestern, southwestern, southeastern, and central regions of the Northern UBD, respectively. The YDW, YDC, and YDE represent the aeolian sand samples from the western, central, and eastern regions of the Eastern YD, respectively.

**Figure 1:**
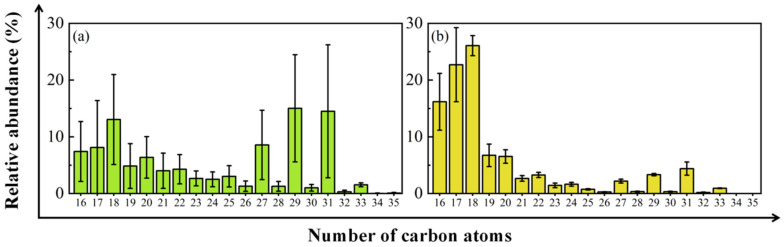
Average desert aeolian sand *n*-alkane distribution patterns. (**a**) The Northern UBD; (**b**) the Eastern YD.

Aeolian sand *n*-alkanes of the Northern UBD and Eastern YD have a total abundance ranging from 0.05 to 0.92 μg/g with an average and standard deviation of 0.25 ± 0.29 (*n* = 8) μg/g; among which, short-chain *n*-alkanes account for 7–79%, and long-chain *n*-alkanes account for 9–79% (Table 1). The aeolian sand long-chain *n*-alkanes of the Northern UBD and Eastern YD are mostly dominated by *n*-C_29_ and *n*-C_31_, and the UBD has a higher average relative abundance of *n*-C_29_, whereas the YD has a higher average relative abundance of *n*-C_31_ (Figure 1).

The UBD aeolian sand has a larger *n*-alkane total abundance and a broader range of *n*-alkane abundance variations, ranging from 0.05 to 0.92 μg/g with an average and standard deviation of 0.29 ± 0.37 μg/g; among which, short-chain *n*-alkanes account for 7–72%, and long-chain *n*-alkanes account for 9–79% (Table 1). Conversely, the YD exhibits a more stable abundance of *n*-alkanes, albeit at a relatively lower level, ranging from 0.11 to 0.24 μg/g with an average and standard deviation of 0.18 ± 0.06 μg/g; among which, short-chain *n*-alkanes have a considerable high relative abundance of 76–79%, while long-chain *n*-alkanes make up only 10–13% (Table 1).

**Figure 2:**
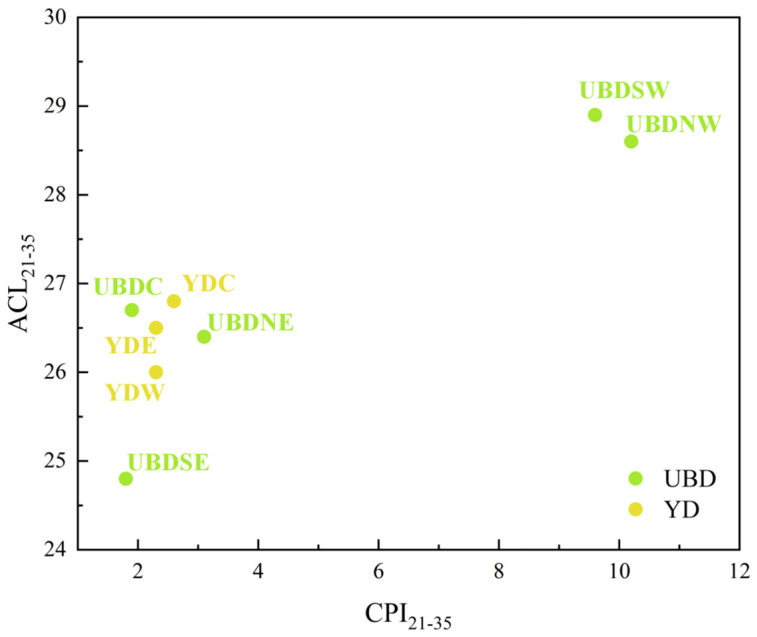
Scatter plot of ACL_21–35_ versus CPI_21–35_ of the aeolian sand samples in the Northern UBD and Eastern YD.

The range, average, and standard deviation of ACL_21–35_ and CPI_21–35_ of aeolian sand from the Northern UBD is 24.8–28.9, 27.1 ± 1.7, and 1.8–10.2, 5.3 ± 4.2, respectively, while those are 26.0–26.8, 26.4 ± 0.4, and 2.3–2.6, 2.4 ± 0.2 for the Eastern YD (Figure 2). The western regions of the Northern UBD (UBDNW and UBDSW) exhibit the highest ACL_21–35_ and CPI_21–35_ among all the aeolian sand samples, while the central and eastern regions of the Northern UBD (UBDC, UBDNE, and UBDSE) bear resemblance to the Eastern YD (YDW, YDC, and YDE), sharing comparatively lower ACL_21–35_ and CPI_21–35_, and UBDSE exhibits the lowest ACL_21–35_ and CPI_21–35_ among all the aeolian sand samples (Figure 2).

### 2.2. Leaf Wax n-Alkane Abundances and Distribution Patterns

**Figure 3:**
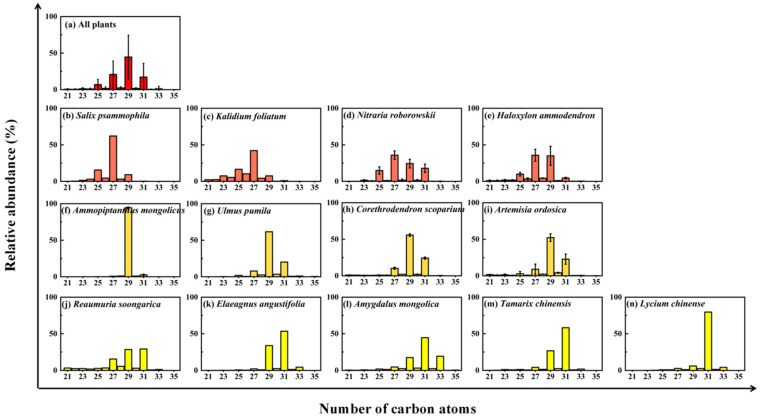
Average *n*-alkane distribution patterns of plant leaf waxes in the Northern UBD. (**a**) All plants, (**b**) *S. psammophila*, (**c**) *K. foliatum*, (**d**) *N. roborowskii*, (**e**) *H. ammodendron*, (**f**) *A. mongolicus*, (**g**) *U. pumila*, (**h**) *C. scoparium*, (**i**) *A. ordosica*, (**j**) *R. soongarica*, (**k**) *E. angustifolia*, (**l**) *A. mongolica*, (**m**) *T. chinensis*, and (**n**) *L. chinense*.

The ΣALK, C_MAX_, ACL_21–35_, and CPI_21–35_ of desert plant leaf wax *n*-alkanes are calculated and shown in Table 2. The abundances and distribution patterns of plant leaf wax *n*-alkanes (*n*-C_21_–*n*-C_35_) in the Northern UBD exhibit distinct differences among various desert plant species and when compared to aeolian sands (Figure 1 and Figure 3). Plant leaf wax *n*-alkanes have a total abundance ranging from 35 to 10,111 μg/g with an average and standard deviation of 1882 ± 3162 (*n* = 33) μg/g (Table 2). Leaf wax *n*-alkanes of *N. roborowskii*, *K. foliatum*, and *S. psammophila* are dominated by *n*-C_27_, while those of *H. ammodendron* are dominated by *n*-C_27_ and *n*-C_29_; those of *A. mongolicus*, *U. pumila*, *C. scoparium*, and *A. ordosica* are dominated by *n*-C_29_; those of *R. soongarica* are dominated by *n*-C_29_ and *n*-C_31_; and those of *E. angustifolia*, *A. mongolica*, *T. chinensis*, and *L. chinense* are dominated by *n*-C_31_, respectively (Table 2). Notably, the leaf wax *n*-alkanes of all desert plant species have odd-over-even *n*-alkane predominance, a characteristic common to most terrestrial vascular plants, as observed by various studies [1,29,33,34,35,36,37], except for *A. mongolicus*.

*A. mongolicus* stands out with the highest total abundance of leaf wax *n*-alkanes among the 13 desert plant species, ranging from 2452 to 10,111 μg/g with an average and standard deviation of 7383 ± 2742 μg/g (Table 2). *L. chinense*, *S. psammophila*, and *E. angustifolia* contain relatively high levels of leaf wax *n*-alkanes (>1000 μg/g, Table 2). In contrast, *A. mongolica*, *N. roborowskii*, *T. chinensis*, *U. pumila*, *C. scoparium*, *A. ordosica*, and *K. foliatum* contain relatively lower levels of leaf wax *n*-alkanes (ranging from 100 to 1000 μg/g, Table 2), and *H. ammodendron* and *R. soongarica* exhibit the least abundance of leaf wax *n*-alkanes (<100 μg/g, Table 2).

**Figure 4:**
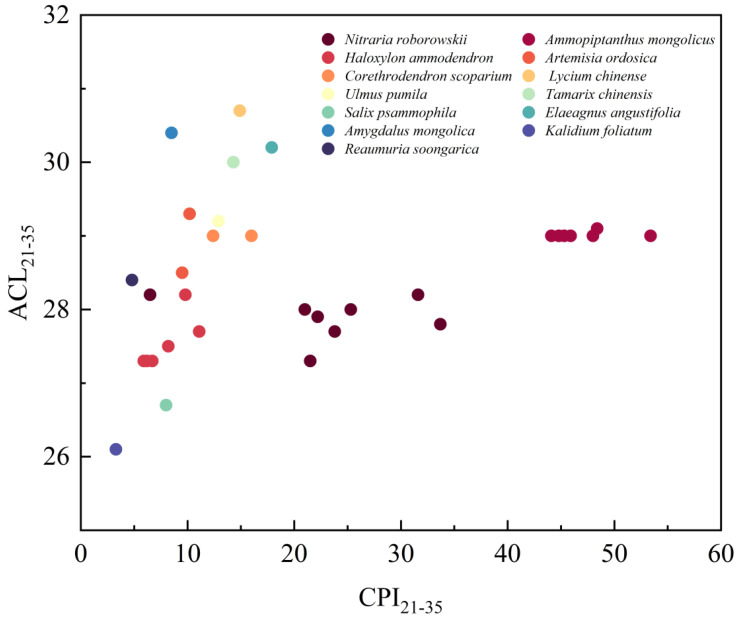
Scatter plot of ACL_21–35_ versus CPI_21–35_ of plant leaf *n*-alkanes from the Northern UBD.

The range, average, and standard deviation of ACL_21–35_ and CPI_21–35_ of plant leaf wax *n*-alkanes of the Northern UBD are 26.1–30.7, 28.5 ± 1.1, and 3.3–53.4, 21.1 ± 15.6, respectively (Figure 4). *L. chinense*, *A. mongolica*, *E. angustifolia*, and *T. chinensis* have relatively high levels of ACL_21–35_ (>30.0, Figure 4), while *S. psammophila* and *K. foliatum* have relatively low levels of ACL_21–35_ (<27.0, Figure 4). *A. mongolicus* has the largest CPI_21–35_ among all of the 13 desert plant species (>40.0, Figure 4), while *R. soongarica* and *K. foliatum* have the lowest levels of CPI_21–35_ (<5.0, Figure 4).

## 3. Discussion

### 3.1. Biogenic Sources of Sedimentary n-Alkanes in the Eastern YD and Northern UBD

The *n*-alkanes with varying carbon chain lengths in sediments typically serve as indicators of specific biological origins: short-chain *n*-alkanes (*n*-C_16_–*n*-C_20_) are normally derived from aquatic organisms like phytoplankton and algae, as well as from microorganisms such as bacteria [38,39,40,41,42,43], and middle-chain *n*-alkanes (*n*-C_21_–*n*-C_26_) are primarily derived from aquatic submerged or floating macrophytes or moss living in palustrine/peat conditions [13,44,45,46,47,48,49], while long-chain *n*-alkanes (*n*-C_27_–*n*-C_35_) are commonly derived from emergent and terrestrial higher plants [1,33,34,50,51].

Although deserts are typically regarded as having minimal microbial activity, the biological soil crusts (BSGs), which extensively cover the plant interspace in deserts, are primarily composed of microorganisms such as cyanobacteria, algae, lichens, mosses, bacteria, and fungi [52]. These BSGs are found to be rich in short-chain *n*-alkanes [53,54,55,56,57]. Hence, short-chain *n*-alkanes in aeolian sands are primarily derived from these microorganisms within the BSGs. Most middle-chain *n*-alkanes in aeolian sands are also likely to be derived from mosses within the BSGs or aquatic macrophytes in nearby desert lakes. Since higher plants in deserts produce minor amounts of middle-chain *n*-alkanes (Figure 3), a small fraction of those in aeolian sand may also be derived from desert higher plants. Meanwhile, long-chain *n*-alkanes in aeolian sand are primarily derived from terrestrial higher plants in deserts (Figure 3).

**Figure 5:**
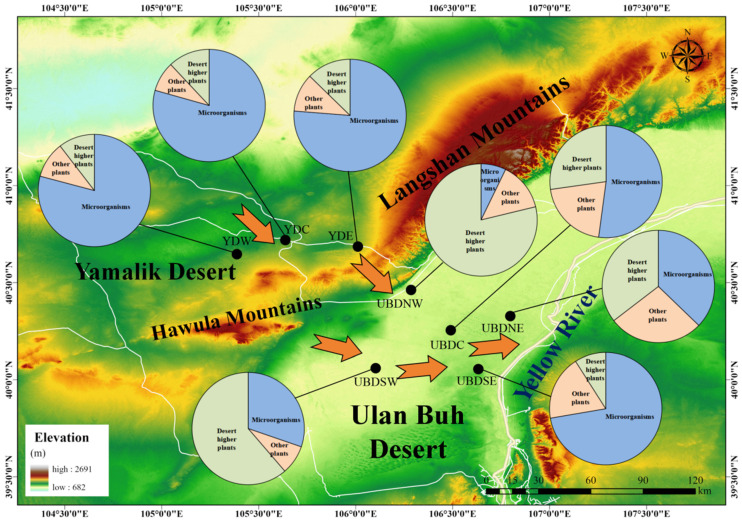
Schematic diagram of the spatial variability of biogenic sources (microorganisms, desert plants, and other plants) for sedimentary *n*-alkanes of Eastern YD and Northern UBD. Yellow arrows signify the trends of sand transport [58].

Figure 5 shows the spatial variability of biogenic sources—encompassing microorganisms, desert plants, and other vegetation—for sedimentary *n*-alkanes in the Eastern YD and Northern UBD, which reveals distinct patterns. Across all regions of the Eastern YD (YDW, YDC, and YDE), the primary biogenic sources of sedimentary *n*-alkanes are microorganisms. In contrast, within the western region of the Northern UBD (UBDNW and UBDSW), the main biogenic sources are desert higher plants. However, in the central (UBDC) and eastern (UBDNE and UBDSE) regions, there is a notable increasing trend of sedimentary *n*-alkanes derived from microorganisms. This spatial variability in the biogenic sources of sedimentary *n*-alkanes clearly illustrates the diverse influences on the depositional processes of *n*-alkanes across different regions of the Eastern YD and Northern UBD.

### 3.2. Decoupling Distribution of n-Alkanes in Aeolian Sand and Surrounding Vegetation

**Figure 6:**
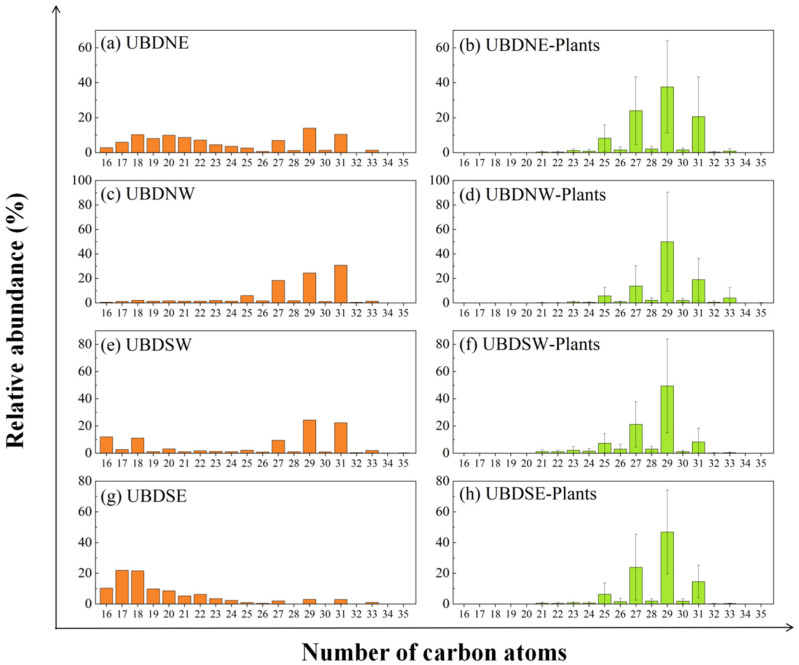
Distribution patterns variations of *n*-alkanes between aeolian sand and the surrounding (<20 km) desert plants in the Northern UBD. (**a**) UBDNE, (**b**) average of plants surrounding UBDNE, (**c**) UBDNW, (**d**) average of plants surrounding UBDNW, (**e**) UBDSW, (**f**) average of plants surrounding UBDSW, (**g**) UBDSE, and (**h**) average of plants surrounding UBDSE.

The *n*-alkane distribution patterns across five aeolian sand samples (which correspond to the NE, NW, SW, SE, and central (C) regions of the Northern UBD) are compared to the surrounding desert plants sampled within a 20 km radius of the aeolian sand sampling sites in Figure 6. The dominant *n*-alkane homolog of desert plants across the NE, NW, SE, and SW regions is *n*-C_29_. This dominance is attributed to the widespread presence of *A. mongolicus* in these regions, which contains exceptionally high levels of *n*-C_29_ (Figure 3). However, the *n*-C_29_ homolog does not consistently predominant long-chain *n*-alkanes in aeolian sand across the Northern UBD, and the *n*-C_31_ homolog shows high concentrations in both the NW and SW regions (Figure 6). This pattern could be attributed to a higher proportion of desert plants like *E. angustifolia*, *A. mongolica*, *T. chinensis*, and *L. chinense* in these regions, which are dominated by *n*-C_31_ in their *n*-alkanes (Figure 3). Nevertheless, differences are also observed in the *n*-alkane distribution patterns between aeolian sand and the surrounding desert vegetation in the NE and SE regions of the Northern UBD, where elevated concentrations of short-chain *n*-alkanes were observed in the aeolian sands.

**Figure 7:**
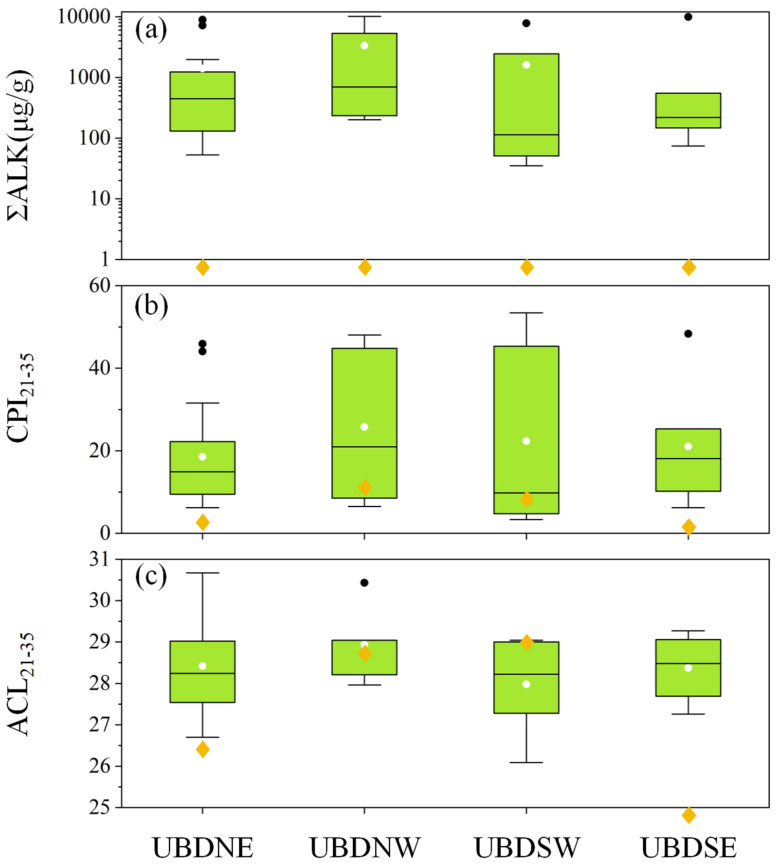
Box plots of leaf wax *n*-alkanes (**a**) ΣALK, (**b**) CPI_21–35_, and (**c**) ACL_21–35_ of desert plants surrounding the four aeolian sand samples in Northern UBD. Each green box portrays the central 50% of the group values, with the central line indicating the group median. The white point denotes the mean of each group, while the black points identify outliers. The yellow diamonds denote the values of the four aeolian sand samples, and the diamonds beneath the horizontal axis indicate the values are lower than the vertical axis’s minimum. ΣALK is represented using logarithmic coordinates.

The prominent dissimilarity of the distribution patterns between plants and soil *n*-alkanes might be a common phenomenon in deserts [28,29]. Microorganisms within desert BSGs can not only introduce short-chain *n*-alkanes into aeolian sands but also enhance the degradation of *n*-alkanes within these aeolian sands. This process resulted in a decrease in CPI and ACL [14,50,59,60,61,62,63]. These biological activities are the main drivers of the prominent dissimilarity of *n*-alkanes distribution patterns between aeolian sands and the surrounding desert plants. The pronounced disparity between the total abundance of *n*-alkanes in the aeolian sand (UBDNE: 0.05, UBDNW: 0.31, UBDSW: 0.92, and UBDSE: 0.06 μg/g, respectively) and in the surrounding desert plants (UBDNE: 53–8895, UBDNW: 201–10,111, UBDSW: 35–7809, and UBDSE: 74–9944 μg/g, respectively) demonstrates the ubiquitous nature of microbial degradation in the Northern UBD (Figure 7). However, significant differences are observed in CPI_21–35_ and ACL_21–35_ between aeolian sands and the surrounding desert plants across different regions of the Northern UBD (Figure 7). The CPI_21–35_ and ACL_21–35_ of sedimentary *n*-alkanes are notably lower than those of the surrounding desert plants, clearly indicating the effect of microbial degradation, particularly in the NE and SE regions of the Northern UBD (Figure 7). In contrast, the CPI_21–35_ and ACL_21–35_ of sedimentary *n*-alkanes in the NW and SW parts of the Northern UBD align with the range observed in the *n*-alkanes of the surrounding desert plants (Figure 7). This suggests that, despite microbial degradation, the aeolian sand *n*-alkanes in the NW and SW parts of the Northern UBD still preserve the *n*-alkane signatures of their surrounding desert vegetation.

### 3.3. Implications for the Preservation of the Sedimentary n-Alkanes in the North UBD

**Figure 8:**
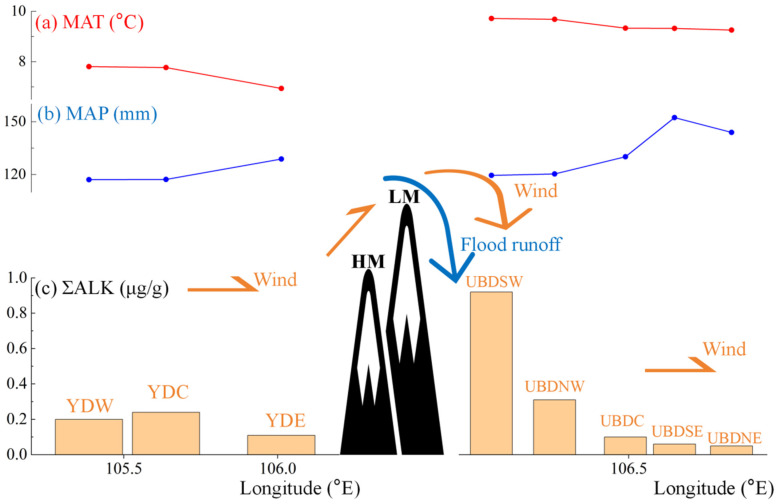
(**a**) Mean annual temperature (MAT) of the Eastern YD and Northern UBD aeolian sand sample sites. MAT data utilize the average values from 1991 to 2020. (**b**) Mean annual precipitation (MAP) of the Eastern YD and Northern UBD aeolian sand sample sites. MAP data utilize the average values from 1991 to 2020. (**c**) Schematic diagram of aeolian sand ΣALK variations along the longitude of the Eastern YD and Northern UBD. Yellow arrows signify the wind direction capable of transporting sand, and blue arrows signify the seasonal flood runoff originating from the Langshan (LM) and Hawula (HM) Mountains.

The spatial variability in the biogenic sources of sedimentary *n*-alkanes, and the divergence in the *n*-alkanes distribution patterns between aeolian sands and the surrounding vegetation, collectively underscore the unique nature of *n*-alkanes sedimentation and preservation in the western region of the Northern UBD (Figure 5, Figure 6 and Figure 7). A significant increase in ΣALK in aeolian sands is also noted in the NW and SW regions of the Northern UBD (Figure 8), which is potentially attributed to multiple environmental factors such as temperature and precipitation. Consequently, we have obtained the MAT and MAP data for the Eastern YD and the Northern UBD from 1991 to 2020. Across all regions of the Northern UBD, the MAT remains relatively stable, ranging from 9 to 10 °C, while the MAT of the Eastern YD is comparatively lower, ranging from 6 to 8 °C (Figure 8). This temperature difference alone does not sufficiently explain the observed increase in ΣALK of the NW and SW regions in the Northern UBD. Precipitation can increase the humidity of deserts and enhance the activity of microorganisms in BSGs [64,65], which, in turn, can accelerate the degradation of sedimentary *n*-alkanes by these microorganisms. The MAP in the C, NE, and SE regions of the Northern UBD is higher than those in the NW and SW regions, which indeed may contribute to the decrease in ΣALK of the C, NE, and SE regions (Figure 8). Nonetheless, the Eastern YD, which has similar low MAP values as the NW and SW regions of the Northern UBD, exhibits notably lower ΣALK values of aeolian sand *n*-alkanes (Figure 8), suggesting that other factors may also influence the preservation and distribution of *n*-alkanes in these areas.

Environmental factors such as water and wind also can play a significant role in the transport of *n*-alkane molecules, which profoundly affect the deposition and variation of *n*-alkanes in local areas [20,21]. Due to the proximity of the LM and HM to the NW and SW regions of the Northern UBD, seasonal gullies originating from these mountains could experience flood runoff during the wet seasons, carrying abundant plant detritus [58,66] and resulting in increased ΣALK values of the NW and SW regions and a higher proportion of *n*-alkanes derived from higher plants. This process indicates the significant role of hydrological dynamics in the transport and deposition of organic compounds in desert environments.

Influenced by the NW winds, the Badain Jaran Desert steadily transfers sand and dust toward the Northern UBD, passing through the YD [58,67,68,69]. This aeolian transport can impact the sedimentary organic matter, including *n*-alkanes, in the Northern UBD, introducing materials from distant sources and altering the local biomarker signatures. The NW winds are transmitted to the E region of the Eastern YD, and due to the obstruction of the LM and HM, they climb upwards and cross the top of the mountain; then, the speed decreases, and the sand and debris carrying will settle in the NW regions of the Northern UBD (Figure 8) [69]. These debris materials brought by the NW winds comprise a mixture of sand and dust, as well as plant debris eroded from the LM and HM (Figure 8). The combined effects of hydraulic and wind forces on the transport of plant-derived materials from LM and HM is the primary cause for the observed increase in the ΣALK and the proportion of *n*-alkanes derived from desert higher plants in the NW and SW regions of the Northern UBD. This highlights the significant impact of environmental dynamics on the distribution and preservation of organic biomarkers in desert ecosystems.

## 4. Materials and Methods

### 4.1. Study Area

**Figure 9:**
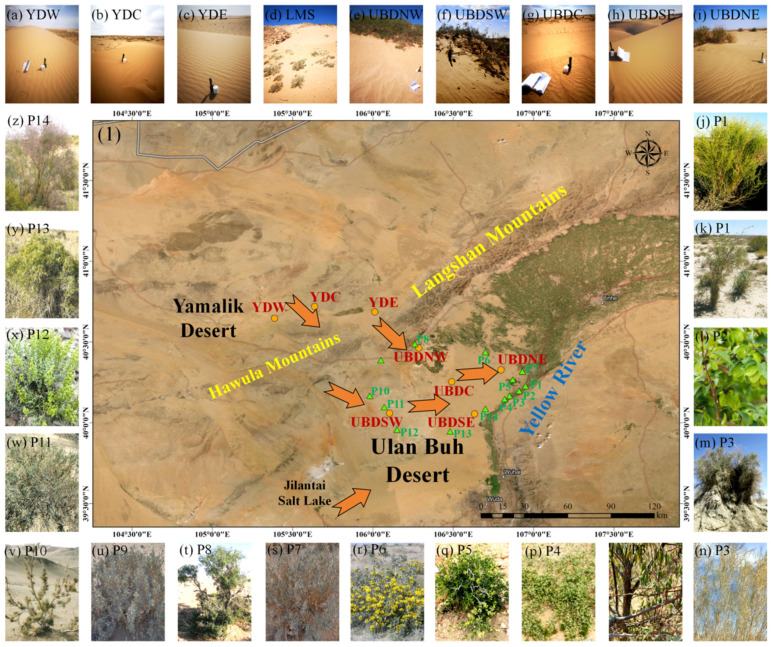
(1) Topographic map depicting the distribution of sampling sites across the study area. Sampling locations for aeolian sands are indicated by yellow dots: YDW (western region of Eastern YD); YDC (central region of Eastern YD); YDE (eastern region of Eastern YD); UBDNW (northwestern region of Northern UBD); UBDSW (southwestern region of Northern UBD); UBDC (central region of Northern UBD); UBDSE (Southeastern region of Northern UBD); UBDNE (northeastern region of Northern UBD). Sampling locations for desert plants are indicated by green triangles: P1–P14. Yellow arrows signify the trends of sand transport [58]. Landscapes of the sampling site: (**a**) shifting dunes of YDW; (**b**) shifting dunes of YDC; (**c**) shifting dunes of YDE; (**d**) a sand cascade at the south slope of the LM; (**e**) shifting dunes of UBDNW; (**f**) shifting dunes of UBDSW; (**g**) shifting dunes of UBDC; (**h**) shifting dunes of UBDSE; (**i**) shifting dunes of UBDNE. Plant photographs of the sampling site: (**j**) *A. ordosica* of P1; (**k**) *L. chinense* of P1; (**l**) *U. pumila* of P2; (**m**) *T. chinensis* of P3; (**n**) *S. psammophila* of P3; (**o**) *E. angustifolia* of P3; (**p**) *N. roborowskii* of P4; (**q**) *N. roborowskii* of P5; (**r**) *A. mongolicus* of P6; (**s**) *A. mongolicus* of P7; (**t**) *A. mongolica* of P8; (**u**) *A. mongolicus* of P9; (**v**) *H. ammodendron* of P10; (**w**) *R. soongarica* of P11; (**x**) *K. foliatum* of P12; (**y**) *N. roborowskii* of P13; (**z**) *C. scoparium* of P14.

The UBD (39.2° N–40.9° N, 105.1° E–107° E) in Northern China is situated in the southwest of the Hetao Plain along the middle reaches of the Yellow River, with the Langshan Mountains (LM) to the north, the Helan Mountains to the south, the Hawula Mountains (HM, also as known as Bayan Urals or Bayanwula Mountains) to the west, the Tengger Desert to the southwest, and bounded by the Yellow River to the east (Figure 9). Recent statistical data indicate that the UBD covers a total area of up to 8980 km^2^, with its overall terrain descending gradually from south to north [58]. The YD, situated in the northwest of the UBD, is a fragmented desert region interspersed within mountain basins and essentially represents the southeastward prolongation of the Badain Jaran Desert [67]. The UBD has an arid climate with the MAP that gradually diminishes from the southeast to the northwest, ranging from 80 to 100 mm with an average value of 105 mm over the past 50 years, while the MAT of the UBD over the past 50 years has been approximately 9.7 °C, exhibiting a pronounced warming trend, ranging from the lowest of −16 °C in the winter to the highest of 33.3 °C in the summer.

Surrounded by mountains on its northern, western, and southern flanks, the UBD presents complex wind conditions (Figure 9). The resultant trend of sand transport in the YD is the southeast, which is in alignment with the movement of local dunes. Due to the barrier of the Helan Mountains, the predominant trend of sand transport in the UBD veers to the northeast at the south of Jilantai Salt Lake. However, the northern region of the UBD experiences a lesser influence from the Helan Mountains, with an overall trend of sand transport towards the east. Although the UBD is the smallest desert in Western China, it boasts a rich variety of dune types, with the shifting dunes making up about 50% of the total area.

The UBD is positioned in a transitional zone between grassland and desert. Here, the vegetation is primarily composed of sand-living, drought-tolerant, and salt-tolerant shrubs and undershrubs that exhibit remarkable adaptability and stress resistance to the local environment [70]. Psammophytes like *Artemisia desertorum* and *Psammochloa villosa* are predominantly found in the shifting dune regions of the Southeastern UBD, and shrubs like *N. roborowskii*, *A. mongolicus*, and *H. ammodendron* are commonly found on the vast lacustrine plains beyond the tall dune regions, and halophytes like *Kalidium* spp. and hydrophytes like *Typha orientalis* are found in the low-lying wetlands of the ancient Yellow River alluvial plain in the Northeast UBD, while *A. ordosica* is primarily found in regions with abundant rainfall in the Eastern UBD [71,72].

### 4.2. Sampling

Our aeolian sand and leaf samples were collected from July 2022 to November 2023, comprising 5 aeolian sand samples from the Northern UBD (UBDNW, UBDSW, UBDC, UBDSE, and UBDNE); 3 aeolian sand samples from the Eastern YD (YDW, YDC, and YDE), and 33 desert plant samples of 10 families, 13 genera, and 13 species from the Northern UBD. All of the sampling sites in this study are given in Figure 9(1), and the landscape and plant photos of representative sampling sites are shown in Figure 9a–z. The sampling locations in the Northern UBD were evenly distributed across the 5 representative regions: NE (northeastern), NW (northwestern), SW (southwestern), SE (southeastern), and C (central), respectively. The 3 YD sampling sites were situated along an east–west transect: W (western), C (central), and E (eastern) in the Eastern YD, separated from the UBD by the LM. Each aeolian sand sample (~1000 g) was collected on the top of sand crests above a depth of about 5 cm within a 50 × 50 cm area as a single sample. Leaf (including assimilative shoot) samples of plant species with a high abundance in the community were collected from more than 5 individual plants. Each leaf sample was carefully stored in a paper bag, ensuring complete air-drying before subsequent preparation.

### 4.3. Extraction and Quantification of n-Alkanes

Leaf material was powdered using a ball mill, while aeolian sand material was ground using an agate mortar. About 60 g of each aeolian sand sample, owing to the scantiness of organic matter, and about 0.1 g of each leaf sample were weighed and placed into their filter paper bags, which were pre-treated using dichloromethane (DCM; Thermo Fisher Scientific (Waltham, MA, USA)) to eliminate organic pollutants. Both aeolian sand and plant samples were Soxhlet-extracted for 48 h with 90 mL DCM/methanol (MeOH; Thermo Fisher Scientific (Waltham, MA, USA)) (9:1, *v/v*). Each total lipid extract was concentrated under a stream of dry N_2_. Non-polar lipids, including *n*-alkanes, were separated from the polar lipids via silica gel column chromatography with 3 mL *n*-hexane (HEX; Thermo Fisher Scientific (Waltham, MA, USA)). The non-polar fraction containing *n*-alkanes was dried under N_2_ and re-dissolved in 300 μL of HEX before the instrumental analysis.

The *n*-alkanes were identified using Shimadzu GC-2010 (Tokyo, Japan) gas chromatography equipped with a DB-5 capillary column (30 m × 0.25 mm × 0.25 μm) and a flame ionization detector (FID). The oven temperature was programmed from 70 to 320 °C at a rate of 3 °C/min and then held at 320 °C for 20 min. The detector temperature was set to 350 °C, and the carrier gas was high-purity nitrogen. The injection was set to 1 μL with a splitless mode. The *n*-alkanes were identified and quantified using the external standard, a known concentration of a homologous series of *n*-C_7_–*n*-C_40_ *n*-alkanes (ANPEL, Shanghai, China). The concentrations of aeolian sand and leaf wax *n*-alkanes were determined by comparing their peak areas to those of the external standard. The concentration of each *n*-alkane was normalized to the dry weight (μg/g) of the aeolian sand and leaf material extracted.

The total concentration of *n*-alkanes (ΣALK), the average chain length (ACL), and the carbon preference index (CPI) were employed to characterize the composition and distribution patterns of the *n*-alkanes. Note that the range of identified *n*-alkanes is generally *n*-C_16_–*n*-C_35_ in aeolian sand samples and *n*-C_21_–*n*-C_35_ in desert plant samples (Figure 1 and Figure 3). To facilitate the comparison, *n*-C_21_–*n*-C_35_ were mostly used in the proxy calculations and discussion. The ΣALK, ACL, and CPI values were calculated as follows [35,73,74,75,76]:

ΣALK_16–35_ = Σ(C_16–35_), (1)

ΣALK_21–35_ = Σ(C_21–35_), (2)

ACL_21–35_ = Σ(n × C_21–35_)/Σ(C_21–35_), (3)

CPI_21–35_ = [Σ_odd_(C_21–33_) + Σ_odd_(C_23–35_)]/[2Σ_even_(C_22–34_)], (4)

where C_n_ represents the concentration of the *n*-alkanes with n carbons.

## 5. Conclusions

We compared *n*-alkane characteristics between aeolian sands and plants of 13 plant species in the Northern UBD, a typical arid desert at the fringe of the East Asian Monsoon region of Northwestern China. A pronounced discrepancy in the *n*-alkane characteristics between aeolian sands and the surrounding desert vegetation is observed in the Northern UBD, indicating that the *n*-alkane biomarkers in aeolian sands are not directly related to the local plant community composition. This suggests that there is a complex relationship between the deposition and preservation of *n*-alkanes. Microbial degradation is the main process causing the decrease in the total abundance of sedimentary *n*-alkanes and the increase in the proportion of short-chain *n*-alkanes, which is mainly controlled by temperature and precipitation in the Northern UBD region as a whole. In the western part of the UBD, due to its special proximity to LM and HM, the influence of local sedimentation processes on sedimentary *n*-alkanes is strengthened. Plant debris transported from LM and HM by seasonal flood runoff and wind leads to the deposition of *n*-alkanes in the western part of the Northern UBD, increasing the total abundance of sedimentary *n*-alkanes and the proportion of long-chain *n*-alkanes.

In summary, the sedimentary *n*-alkane characteristics in the Central and Eastern UBD are a product of both local plant leaf wax *n*-alkanes influenced by external factors such as temperature and precipitation, and those in the Western UBD reflect an additional influence from a more pronounced local sedimentary process. These insights into the temporal and spatial scales of *n*-alkane inputs from desert plants to aeolian sands are vital for the precise interpretation of *n*-alkane biomarkers in paleoclimatic reconstructions within desert settings.

## Figures and Tables

**Table 1:** Abundances of desert aeolian sand *n*-alkanes and related proxies.

Samples	ΣALK (μg/g)	Σ_16–20_/ΣALK (%)	Σ_27–35_/ΣALK (%)	C_MAX_ of Long-Chain *n*-Alkanes	ACL_21–35_	CPI_21–35_
UBDNE	0.05	37	35	29	26.4	3.1
UBDNW	0.31	7	79	31	28.6	10.2
UBDSW	0.92	30	61	29	28.9	9.6
UBDSE	0.06	72	9	29, 31	24.8	1.8
UBDC	0.10	52	27	29	26.7	1.9
YDW	0.20	79	10	29, 31	26.0	2.3
YDC	0.24	79	12	31	26.8	2.6
YDE	0.11	76	13	31	26.5	2.3

**Table 2:** Abundances of plant leaf wax *n*-alkanes and related proxies in the Northern UBD.

Desert Plant Species	ΣALK (μg/g)	C_MAX_	ACL_21–35_	CPI_21–35_
*Salix psammophila*	1572	27	26.7	8.0
*Kalidium foliatum*	114	27	26.1	3.3
*Nitraria roborowskii*	410 ± 168 (201–653) ^1^	27	27.9 ± 0.3 (27.3–28.2)	23.2 ± 8.2 (6.5–33.7)
*Haloxylon ammodendron*	86 ± 32 (51–131)	27, 29	27.5 ± 0.4 (27.3–28.2)	10.4 ± 1.6 (8.3–12.3)
*Ammopiptanthus mongolicus*	7383 ± 2742 (2452–10,111)	29	29.0 ± 0.0 (29.0–29.1)	47.1 ± 3.2 (44.1–53.4)
*Ulmus pumila*	169	29	29.2	12.9
*Corethrodendron scoparium*	154 ± 9 (147–160)	29	29.0 ± 0.0 (29.0–29.0)	14.2 ± 2.6 (12.4–16.0)
*Artemisia ordosica*	152 ± 81 (95–209)	29	28.9 ± 0.6 (28.5–29.3)	9.8 ± 0.5 (9.5–10.2)
*Reaumuria soongarica*	35	29, 31	28.4	4.8
*Elaeagnus angustifolia*	1233	31	30.2	17.9
*Amygdalus mongolica*	697	31	30.4	8.5
*Tamarix chinensis*	209	31	30.0	14.3
*Lycium chinense*	1975	31	30.7	14.9

^1^ Average ± standard deviation (range).

## Data Availability

The data from this study are available upon reasonable request.
